# IARC Carcinogen Update

**DOI:** 10.1289/ehp.113-1280416

**Published:** 2005-09

**Authors:** Marie-Claude Rousseau, Kurt Straif, Jack Siemiatycki

**Affiliations:** INRS-Institut Armand-Frappier, Université du Québec Montréal, Québec, Canada, E-mail: marie-claude.rousseau@iaf.inrs.ca; International Agency for Research on Cancer, Lyon, France, E-mail: straif@iarc.fr; Université de Montréal Montréal, Québec, Canada, E-mail: j.siemiatycki@umontreal.ca

We recently published an article in which we presented a list of occupational carcinogens (Siemiatycki et al. 2004), based on the International Agency for Research on Cancer (IARC) Monographs Program. Our review covered Volumes 1–83 of the *IARC Monographs*. However, because the IARC Monograph Program is ongoing, the list of occupational carcinogens will need to be periodically updated. Since we completed our article, there have been three Monograph meetings that addressed substances that can be classified as occupational; therefore, we would like to notify readers of some important changes in the list of occupational carcinogens. Table 1 shows summary information about occupational substances and mixtures that were recently evaluated by IARC as human carcinogens (group 1), probable human carcinogens (group 2A), or possible human carcinogens (group 2B). As we did in our earlier article (Siemiatycki et al. 2004), we added to the IARC evaluations our assessment of the main occupations in which the agent may be found and the target organ for carcinogenicity.

Volume 86 focuses on cobalt in hard-metals and cobalt sulfate, gallium arsenide, indium phosphide, and vanadium pentoxide (IARC, in press a) In our article (Siemiatycki et al. 2004), cobalt and cobalt compounds were listed as Group 2B human carcinogens. In IARC’s recent evaluation (IARC, in press a), cobalt metal with tungsten carbide is classified in Group 2A, whereas cobalt metal without tungsten carbide, cobalt sulfate, and other soluble cobalt(II) salts remain in Group 2B. Three substances for which there were no previous IARC evaluations have now been evaluated and classified: gallium arsenide is classified as a Group 1 human carcinogen, indium phosphide as a Group 2A (probable) human carcinogen, and vanadium pentoxide as a Group 2B (possible) human carcinogen (IARC, in press a).

Volume 87 (IARC, in press b) updates the prior evaluations on inorganic and organic lead compounds, which were included in Volume 23 (IARC 1980) and in Supplement 7 (IARC 1987). Previously, lead and inorganic lead compounds were classified in Group 2B, whereas organic lead compounds were classified in Group 3. The most recent IARC evaluation results in an upgrading of inorganic lead compounds to Group 2A; organic lead compounds remain in Group 3 (IARC, in press b). The Working Group, however, noted that part of the organic lead is metabolized into ionic lead, which would be expected to present the same toxicity as inorganic lead.

In Volume 88, formaldehyde was upgraded from a Group 2A (probable) to a Group 1 human carcinogen (IARC, in press c; Cogliano et al. 2005). The other two substances covered by this monograph, 2-butoxyethanol and 1-*tert*-butoxy-2-propanol, are evaluated as Group 3 (not classifiable).

## Figures and Tables

**Table 1:** Substances and mixtures that have been evaluated by IARC as human carcinogens and that are occupational exposures, based on *Monograph* Volumes 84–90.

Substance or mixture	Occupation or industry in which the substance is found^a^	Site(s)	IARC classification	IARC Monograph
Cobalt metal with tungsten carbide	Production of cemented carbides (hard-metal industry); tool grinders; saw filers; diamond polishers	Lung^b^	2A	86
Cobalt metal without tungsten carbide	Miners; production of alloys; processing of copper and nickel ore; glass and ceramic production; welders of cobalt-containing alloys	Uncertain	2B	86
Cobalt sulfate and other soluble cobalt(II) salts	Electroplating and ceramic industries	Uncertain	2B	86
Gallium arsenide	Production; microelectronics industry (integrated circuits and optoelectronic devices)	Uncertain	1^c^	86
Indium phosphide	Production; microelectronics industry (integrated circuits and optoelectronic devices)	Uncertain	2A^d^	86
Vanadium pentoxide	Ore refining and processing; chemical manufacturing industry; maintenance of oil-fired boilers and furnaces	Uncertain	2B	86
Inorganic lead compounds	Lead smelters; plumbers; solderers; occupations in battery recycling smelters; production of lead-acid batteries; printing press occupations; pigment production; construction and demolition	Lung^b^ Stomach^b^	2A	87
Formaldehyde	Production; pathologists; medical laboratory technicians; plastics; textile and plywood industry	Nasopharynx^e^ Leukemia^b^ Nasal sinuses^b^	1	88

a Not necessarily an exhaustive list of occupations/industries in which this agent is found; not all workers in these occupations/industries are exposed. The term “production” is used to indicate that this substance is man-made and that workers may be exposed in the production process.

b We judged that the evidence for an association with this site was suggestive.

c In reaching an overall evaluation of Group 1, the working group noted the potential for gallium arsenide to cause cancer through releases of a small amount of its arsenic, which behaves as inorganic arsenic at the sites where it is distributed. Arsenic and arsenic compounds have been evaluated as IARC Group 1, carcinogenic to humans. For arsenic in drinking water, the most recent IARC evaluation of arsenic [Volume 84; (IARC 2004)], there was sufficient evidence in humans that arsenic causes cancers of the urinary bladder, lung, and skin; the evidence for cancers of the liver and kidney was limited.

d Absence of data on cancer in humans; the final evaluation for carcinogenicity was upgraded from 2B to 2A based on evidence of carcinogenicity in experimental animals.

e The evidence was sufficient.

## References

[b1-ehp0113-a00580] Cogliano VJ, Grosse Y, Baan RA, Straif K, Secretan MB, El Ghissassi F, the Working Group for Volume 88 (2005). Summary of *IARC Monographs* on Formaldehyde, 2-Butoxyethanol, and 1-*tert*-Butoxy-2-Propanol. Environ Health Perspect.

[b2-ehp0113-a00580] IARC (1980). Some Metals and Metallic Compounds. IARC Monogr Eval Carcinog Risk Chem Hum.

[b3-ehp0113-a00580] IARC 1987. Overall Evaluations of Carcinogenicity: An Updating of IARC Monographs Volumes 1 to 42. IARC Monogr Eval Carcinog Risk Chem Hum(suppl 7). 3482203

[b4-ehp0113-a00580] IARC (2004). Some Drinking-Water Disinfectants and Contaminants, Including Arsenic. IARC Monogr Eval Carcinog Risk Hum.

[b5-ehp0113-a00580] IARC In press a. Cobalt in Hard-metals and Cobalt Sulfate, Gallium Arsenide, Indium Phosphide and Vanadium Pentoxide. IARC Monogr Eval Carcinog Risks Hum 86. PMC4781610 16906675

[b6-ehp0113-a00580] IARC In press b. Inorganic and Organic Lead Compounds. IARC Monogr Eval Carcinog Risks Hum 87. PMC4781642 17191367

[b7-ehp0113-a00580] IARC In press c. Formaldehyde, 2-Butoxyethanol and 1-*tert*-Butoxy-2- propanol. IARC Monogr Eval Carcinog Risks Hum 88. PMC4781641 17366697

[b8-ehp0113-a00580] Siemiatycki J, Richardson L, Straif K, Latreille B, Lakhani R, Campbell S (2004). Listing occupational carcinogens. Environ Health Perspect.

